# QRS as a Risk Stratification Tool: Putting the Fragments Together

**DOI:** 10.1016/s0972-6292(16)30709-4

**Published:** 2014-01-01

**Authors:** Anandaraja Subramanian

**Affiliations:** Indira Gandhi Government General Hospital and Postgraduate Institute, Pondicherry, India.

**Keywords:** Fragmented QRS, risk stratification

Implantable cardioverter defibrillator (ICD) is the currently accepted treatment for prevention of sudden cardiac death (SCD). Left ventricular ejection fraction is the only useful tool currently available to risk stratify patients at risk of SCD. Several other parameters have been studied for further risk stratification of SCD. Selection of appropriate subjects will improve the efficacy of treatment and also avoid unwanted ICD implantations in low risk patients. Role of QRS duration, QT dispersion, signal-averaged ECG, heart rate variability, ambulatory ECG monitoring, heart rate turbulence, T wave alternans, baroreceptor sensitivity and heart rate recovery have been investigated to identify patients at higher risk of arrhythmia and sudden cardiac death. However, none of these non-invasive tests could reliably stratify patients at risk of sudden cardiac death [1].

Fragmented QRS complex (fQRS) has been identified as one of the non-invasive markers of mortality and sudden cardiac death [2]. fQRS has been defined as the presence of additional R wave or notching in the nadir of the S wave in two contiguous leads corresponding to a major coronary artery territory in the resting 12 lead ECG. Wide fQRS (QRS duration >120 ms) has been defined as various RSR patterns with or without a Q wave, with more than 2 R waves or more than 2 notches in the R wave or more than 2 notches in the upstroke or downstroke of the S wave, in two contiguous leads corresponding to a major coronary artery territory. Slow and inhomogenous conduction through the diseased myocardium and conduction system is the mechanism of fQRS [3,4]. Various studies have correlated the presence of fQRS in predicting myocardial scars [5,6], poor outcomes [7] and ventricular arrhythmias [8-10].

In this issue of the journal Apiyasawat et al [11] have reported the role of fQRS in predicting appropriate cardioverter-defibrillator therapy. There was a strong association between fQRS and ventricular arrhythmic events. fQRS was associated with more than five times the chance of receiving appropriate therapy. Similarly Das et al [12] in their study of 361 patients reported that the event free survival was significantly lower in patients with fQRS. However in an analysis of MADIT II study [13], fQRS was not associated with risk of sudden or all cause mortality. Similarly studies by Cheema et al [14] and Forleo et al [15] also failed to show an association between fQRS and arrhythmic events in patients with AICD. These differences can be attributed to different etiology (structural vs. non structural), substrates (ischemic vs. non ischemic), indications (primary vs. secondary), duration of QRS (normal vs. wide), location of fQRS (anterior vs. inferior vs. lateral) and extent of fQRS (single vs. more than one territory).

Thus based on the available data in the literature, fQRS cannot be reliably used for risk stratification of SCD currently. More understanding is needed on the relation between fQRS, scar burden and arrhythmia inducibility [16] before it can be routinely used for risk stratification. Also more objective and quantitative methods of evaluating fQRS will help in appropriate assessment of this tool [17]. Until then we have to continue to use ejection fraction and NYHA functional class for risk stratification in patients with structural heart disease.
